# Aging insights from heterochronic parabiosis models

**DOI:** 10.1038/s41514-024-00166-0

**Published:** 2024-08-17

**Authors:** Francisco Alejandro Lagunas-Rangel

**Affiliations:** Department of Surgical Sciences, Uppsala University, Uppsala, Sweden

**Keywords:** Senescence, Transcription

## Abstract

Heterochronic parabiosis consists of surgically connecting the circulatory systems of a young and an old animal. This technique serves as a model to study circulating factors that accelerate aging in young organisms exposed to old blood or induce rejuvenation in old organisms exposed to young blood. Despite the promising results, the exact cellular and molecular mechanisms remain unclear, so this study aims to explore and elucidate them in more detail.

## Introduction

Aging is the result of the accumulation of a wide range of molecular and cellular damage over time, leading to a gradual decline in physical and mental capacity, increased vulnerability to the development of pathologies such as cancer, diabetes, cardiovascular disorders, neurodegenerative and infectious diseases, among others, as well as the risk of death^1–3^. Hallmarks of aging include genomic instability, telomere attrition, epigenetic alterations, loss of proteostasis, impaired macroautophagy, dysregulation of nutrient sensing, mitochondrial dysfunction, cellular senescence, stem cell depletion, altered intercellular communication, chronic inflammation and dysbiosis^4^.

Parabiosis is a surgical procedure that joins the circulatory systems of two living organisms of the same species. Its name comes from the Greek words “para” (next to) and “bios” (life)^5^. First introduced by French physiologist Paul Bert in the 1860s to advance organ transplantation techniques, its usefulness was later extended to aging research^6^. Likewise, although originally performed on white albino rats, mice have become the main subjects of this procedure.

Parabiosis has shown great potential to provide clues about the fundamental mechanisms of aging and how lifespan could be increased. For this purpose, the circulatory systems of two animals of different ages are fused. During heterochronic parabiosis, old animals can significantly extend their lifespan by gaining better protection against fatal diseases or treatments that would otherwise lead to premature mortality. In contrast, young mice show deleterious effects when exposed to the systemic milieu of their older counterparts. Thus, heterochronic parabiosis models serve as valuable tools for examining the influence of circulating factors in the bloodstream on various aging-related processes^7^.

The connection established through heterochronic parabiosis facilitates the exchange of vital blood components such as hormones, enzymes, circulating factors, blood cells, non-coding RNAs, extracellular vesicles (EVs) and even cellular organelles such as functional mitochondria, along with other cellular materials^5,8–10^. This exchange allows researchers to explore the systemic effects of blood factors on aging at the cellular and tissue level, as well as on the development of age-related diseases and other significant parameters associated with aging. As a result, a notable conclusion to be drawn from these studies is the possibility of intervening and potentially reversing several aspects of age-related decline^5^.

It should be noted that two distinct paradigms can be analyzed during heterochronic parabiosis: parabiosis-induced rejuvenation and parabiosis-induced acceleration of aging. The former occurs in the old animal exposed to the systemic milieu of the young animal, whereas the latter occurs in the young animal exposed to the systemic milieu of its old counterpart. Research has shown that almost all cell types possess the capacity to undergo changes in response to alterations in blood composition induced by heterochronic parabiosis, including those not directly exposed to it^11^. In this sense, heterochronic parabiosis has shown remarkable beneficial effects, such as improvements in cognition, muscle strength, cardiac, hepatic and pancreatic function and bone repair in mice^12^. Specifically, the systemic milieu of young individuals has the capacity to reverse epigenetic markers, transcriptomic profiles, proteomic features, and metabolomic signatures associated with aging. In contrast, the systemic milieu of older individuals tends to elicit opposing effects^13^. Furthermore, the involvement of some circulatory proteins has been identified as possible mediators of these effects (Table 1)^14–17^.

**Table 1:** Circulating factors identified by heterochronic parabiosis that influence aging

Organ	Age of mice	Crosslinking time	Factor	Effect	Description	Reference
Brain	2–3 months (young) 20–22 months (old)	5 weeks	CCL11	Detrimental	Trigger pro-oxidant and inflammatory pathways to promote cellular senescence.	^14^
	3 months (young) 23 months (old)	5 days	β2M	Detrimental	Stabilize the interaction of MHC-1 and PIRB to interfere with synaptic refinement. Disrupt NMDA signaling.	^28^
	3 months (young) 20 months (old)	24 days	PF4	Beneficial	Bind to CXR3 to mitigate inflammatory factors.	^50^
	3–4 months (young) 20–22 months (old)	4–5 weeks	HSF1	Beneficial	Induce transcription of heat shock chaperones.	^13,41^
Bone	3 months (young) 21 months (old)	4 weeks	GDF11	Beneficial	Upregulate the SMAD2/3 pathway to promote COL2A1 secretion	^67,68^
Muscle	3 months (young) 21 months (old)	4 weeks			Promote differentiation by activating the SMAD2/3 pathway. Improve mitochondrial activity by increasing PGC1α levels.	^67,88^
Heart	2–3 months (young) 21–23 months (old)	4 weeks			Reduce senescent cells, inflammation, and reverse cardiac hypertrophy.	^88,93^
Liver	4 months (young) 20 months (old)	5 weeks	MANF	Beneficial	Decrease inflammation.	^87^
	3–4.5 months (young) 19 months (old)	5 weeks	miR-29c-3p	Detrimental	Target extracellular and secretory proteins	^8^
Eye	3 months (young) 11 months (old)	4 months	TPM1	Beneficial	Regulate inflammatory responses	^102^

All the referenced studies used mice of the C57BL/6 strain.

Notably, despite these promising findings, the precise mechanisms underlying the functioning of heterochronic parabiosis at the cellular and molecular level remain incompletely understood. Hence, the aim of this study was to provide an overview of the main effects observed in models of heterochronic parabiosis in the context of aging and its associated diseases. In addition, special attention was paid to the investigation of the molecular interactions and pathways responsible for producing these effects in order to gain a deeper understanding of this phenomenon. Unraveling these intricate mechanisms is essential to understand the complexity of this phenomenon and to develop strategies aimed at delaying the deleterious effects of aging, ultimately improving our quality of life.

## General aspects of heterochronic parabiosis

One study indicated that out of a total of 20 tissues and 122,280 cells, approximately 49 cell types are susceptible to the accelerated aging effects of parabiosis, while 51 cell types are susceptible to induced rejuvenation^11^. Circulating factors present in the blood of elderly subjects have been found to accelerate the typical changes of aging, contributing to various age-related processes. In contrast, the blood of young individuals possesses a remarkable rejuvenating potential, capable of reversing age-related profiles. Specifically, adipose mesenchymal stromal cells, hematopoietic stem cells and hepatocytes stand out as cell types that show increased sensitivity to the effects of parabiosis-induced rejuvenation and parabiosis-accelerated aging. At the pathway level, studies have indicated that young blood not only reverses established aging patterns, but also triggers the activation of new sets of genes. For example, in parabiosis-induced rejuvenation, there is evidence of enhanced mitochondrial function, as evidenced by the complete rescue of genes encoding subunits of the electron transport chain^11,13^. Similarly, it has been observed that many cell-cell communication networks, which are disrupted during the aging process, undergo alterations in response to heterochronic parabiosis^13^.

Circulating small extracellular vesicles (EVs) from young mice increase peroxisome proliferator-activated receptor gamma coactivator 1α (PGC1α) expression in aged mice through their miRNA cargoes, including miR-144-3p, miR-149-5p and miR-455-3p. These miRNAs target amyloid-β precursor protein (APP), poly[ADP-ribose] polymerase 2 (PARP2), and hypoxia-inducible factor 1α inhibitor (HIF1AN), respectively, which result in improved mitochondrial function and alleviates mitochondrial deficits in aged tissues^10^.

Heterochronic parabiosis decreases senescent cell levels and prevents the inhibition of senescent cell elimination in old mice exposed to a young systemic milieu. Conversely, it increases senescent cell levels in young mice exposed to a young systemic milieu^18^. In preclinical models of aging, the accumulation of senescent cells is associated with a number of chronic diseases, geriatric syndromes, multimorbidity and accelerated aging. In contrast, the reduction of these cells by genetic and/or pharmacological methods has been shown in animal studies to prevent, delay and alleviate various aging-related diseases and their effects^19^. Expression of markers of the senescence-associated secretory phenotype (SASP) factors, including cyclin-dependent kinase inhibitor 1 (CDKN1A), cyclin-dependent kinase inhibitor 2A (CDKN2A), C-C motif chemokine 2 (CCL2) and proinflammatory cytokines such as interleukin-1β (IL1B), interleukin-6 (IL-6) and tumor necrosis factor α (TNFα), was significantly reduced in multiple tissues of old mice exposed to a young environment. In contrast, senescence markers were simultaneously increased in heterochronic young parabionts^20^. Recent research has shown that the removal of senescent immune cells significantly improves the immune function of old animals. By eliminating these cells, the animals prevent the release of cytokines that promote chronic inflammation and their immune cells respond more effectively to infectious agents and tumor cells, similar to the responses observed in young individuals. This improvement not only results in a higher quality of life, but also extends their lifespan^21–23^.

The aging process often involves intricate alterations in iron metabolism, such as reduced serum iron levels and increased tissue iron deposition in various tissues of aged mice. Surprisingly, exposure of old animals to the systemic milieu of young mice has been observed to reverse these age-related changes. Correlation analysis further underscores the importance of iron metabolism in the aging process, revealing a negative correlation between tissue iron levels and telomerase expression in vital organs such as liver, kidney and heart of parabiotic mice^24^.

Accelerated aging has been reported to induce more cell-specific changes, reflecting divergent pathways in different cell types. In contrast, induced rejuvenation involves a more coordinated process, suggesting a conserved response in various cell types^11^. Currently, it is uncertain whether the beneficial effects observed in older individuals during heterochronic parabiosis are of the same level as the detrimental effects observed in younger individuals exposed to this same procedure. In this context, there is controversy about whether the negative effects of accelerated aging outweigh the positive effects of induced rejuvenation. Some studies support this assertion^25–29^, while others have reported contradictory findings^30^. In addition, some studies indicate that the result may vary depending on the cell type^11^. It is also crucial to consider the possible influence of other factors and experimental differences that have contributed to the conclusions reached in these studies.

Furthermore, it has been mentioned that short-term parabiosis results in smaller and less enduring effects compared to long-term parabiosis. Following the separation of the individuals, long-term parabiosis showed more pronounced and sustained effects over time^31^. Similarly, it has been mentioned that the magnitude of induced rejuvenation or accelerated aging is positively correlated with the age difference between mice. Apparently, the greater the age difference, the greater the observed benefits or detriments^32^. Although this phenomenon does not occur in all cases. For example, although statistically significant changes in certain SASP factors were observed in the liver of 67-week-old mice paired with their 4-week-old and 8-week-old counterparts, it is not definitively demonstrated that older mice paired with 4-week-old animals experienced more pronounced overall parabiosis-induced rejuvenation^33^. Furthermore, the effects of parabiosis-accelerated aging may be exacerbated by various stressors, such as traumatic surgery and infectious diseases^34^.

## Impacts of heterochronic parabiosis across different organs

### Brain

**Fig. 1:**
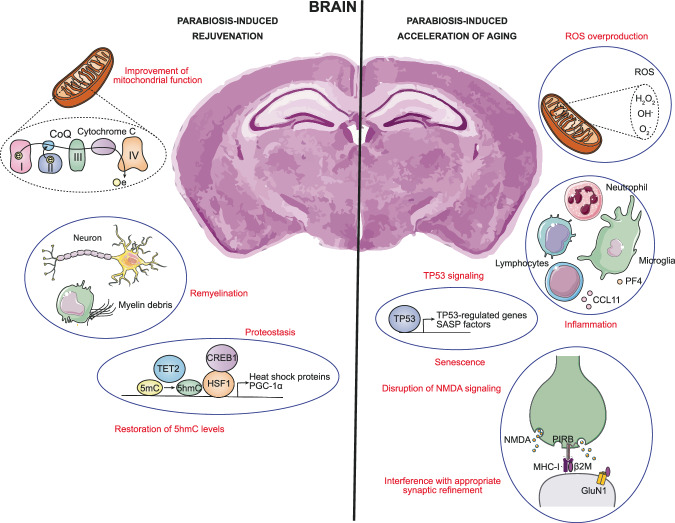
Effects observed in the brain during heterochronic parabiosis. From the perspective of parabiosis-induced rejuvenation, improvements in mitochondrial activity, neuronal remyelination, and mechanisms supporting proteostasis are observed. In contrast, in the context of parabiosis-induced accelerated aging, there is increased ROS production, inflammation, activation of TP53 signaling leading to induction of senescence, disruption of NMDA signaling, and interference with synaptic refinement.

Heterochronic parabiosis causes significant alterations in several brain cell types, including neural stem cells, neural progenitor cells ependymal cells, choroid plexus epithelial cells, GABAergic neurons, glutamatergic neurons, neuroendocrine cells, microglia, oligodendrocytes and arachnoid barrier cells^11,13,32^. This allows researchers to analyze both accelerated aging and induced rejuvenation paradigms to understand different regulatory patterns within various critical ontologies (Fig. 1).

In a study focused on investigating the neurogenic region of the subventricular zone, mice exposed to young blood showed a significant rejuvenating effect on activated neural stem cells and neural progenitor cells. This led to an average reduction of 4.52 months in chronological age and 2.51 months in biological age, as determined by a cell type-specific aging clock. In contrast, young mice exposed to old blood experienced an increase in predicted chronological age in several brain cell types, confirming the detrimental impact of old blood on other tissues^32^. Chronological age refers to the time of an individual’s existence, while biological age refers to the age of its cells^35^. In other words, chronological age denotes the passage of time since birth, while biological age reflects the state and function of the body’s cells, which does not always correspond to the passage of time.

In particular, pathways associated with mitochondrial activity, including oxidative phosphorylation and the electron transport chain, decreased during parabiosis-accelerated aging and increased during parabiosis-induced rejuvenation^13^. Since mitochondria are the powerhouse of the cell and perform other crucial cellular functions such as regulating apoptosis, maintaining calcium levels, and managing reactive oxygen species (ROS) and redox signaling, their decline in quality and activity are a major hallmark of aging^36^. This indicates that certain factors present in the blood of young animals, which are lost during the aging process, can effectively reverse the functional decline of mitochondria.

Furthermore, pathways linked to homeostasis and oxidative stress metabolism demonstrate a similar pattern of increase during parabiosis-accelerated aging^13^. In this regard, as a consequence of mitochondrial dysfunction and other aspects of aging, an increase in ROS levels occurs. When this increase exceeds the inherent antioxidant defenses of the cell, an imbalance is created, resulting in oxidative stress that can cause molecular and cellular damage^37^.

Specifically in brain endothelial cells, the parabiosis-induced rejuvenation process was found to involve down-regulation of inflammatory pathways, such as tumor necrosis factor α (TNFA) signaling through nuclear factor κB (NF-κB)^13^. It is well known that endothelial cells, which are part of the neurovascular unit along with glial cells and pericytes, are vital for nutrient supply, waste removal and blood-brain barrier formation. With aging, endothelial senescence can initiate an inflammatory cycle, alter brain homeostasis and contribute to cognitive decline^38^.

Short-term exposure to young systemic factors produces both functional and structural rejuvenation of the cerebral microcirculation. Old mice exposed to blood from young animals show a significant increase in cortical capillary density and a restoration of blood-brain barrier integrity. Meanwhile, young mice exposed to blood from older animals rapidly develop opposite effects, which could promote age-related cerebromicrovascular pathologies, such as microvascular amyloid deposition and increased microvascular fragility^39^.

Additionally, a reduction is noted in the senescence-associated TP53 pathway during brain endothelial cell parabiosis-induced rejuvenation^13^. This indicates that there are fewer conditions causing cell arrest and senescence, possibly as a result of reduced secretion of SASP factors. Furthermore, it suggests that senescent cells are being effectively eliminated as rejuvenation of the immune system response occurs^36^.

In brain endothelial cells, proteostasis-associated pathways, such as heat shock factor 1 (HSF1) activation and de novo protein folding, showed up-regulation during parabiosis-induced rejuvenation and down-regulation during parabiosis-accelerated aging. This trend paralleled a similar pattern observed in the generation of precursor metabolites, as well as in the metabolism of amino acids and their derivatives^13^. Aging alters proteostasis, notably affecting protein folding and proteolytic systems^36^. In addition, there is a reduced responsiveness to anabolic stimuli for protein synthesis, including amino acid synthesis^40^. Specifically, HSF1 acts as a master transcriptional regulator, orchestrating the induction of heat shock protein chaperones to ensure proteome quality control and maintain proteostasis^41^.

In a model of heterochronic parabiosis rejuvenation, hippocampal ten-eleven translocation 2 (TET2) expression was restored, leading to increased levels of 5-hydroxymethylcytosine (5hmC) associated with neurogenic processes^42^. 5hmC is an epigenetic mark mainly involved in the positive regulation of gene expression and patients with neurodegenerative disorders, such as Alzheimer’s disease, Parkinson’s disease and Huntington’s disease, its levels are significantly compromised. Therefore, restoration of 5hmC levels by parabiosis helped to mitigate the pronounced age-related decline in neurogenesis, resulting in improved learning and memory abilities^42^.

In addition to brain endothelial cells, other brain cell types such as microglia and neuroblasts also showed a rejuvenating trend induced by parabiosis. However, the observed effects were less uniform or consistent across subjects^32^. In a preprint, hippocampal microglia from young heterochronic parabionts have been reported to show reduced expression of homeostatic genes, along with increased expression of ribosomal genes, inflammatory activation genes, and myeloid activation genes^43^. In old mice exposed to a young systemic milieu, remyelination is markedly enhanced. This enhancement primarily involves the mobilization of blood-derived monocytes from the younger parabiotic partner to repair sites in the old counterpart, where they play a crucial role in clearing myelin debris. Consequently, this process promotes angiogenesis at demyelinated sites, encouraging the proliferation of oligodendrocyte precursor cells. It also reverses the age-related hindrance in the differentiation of these cells, thus restoring their ability to mature into functional oligodendrocytes^44^.

Exposure of a young mouse to an old systemic milieu has been shown to decrease synaptic plasticity, impair contextual fear conditioning, and impair spatial learning and memory. Further in this area, it was identified that levels of certain chemokines, including CCL2, C-C motif chemokine ligand 11 (CCL11), C-C motif chemokine ligand 12 (CCL12), C-C motif chemokine ligand 19 (CCL19), haptoglobin and β2-microglobulin (β2M), correlate with reduced neurogenesis in heterochronic parabionts and aged mice^14^.

Focusing specifically on CCL11, its levels were found to be elevated in the plasma and cerebrospinal fluid of healthy aging humans. This finding led to the conclusion that the heightened systemic level of CCL11 is, at least in part, associated with the harmful phenomenon observed in young parabiotic animals exposed to the blood of old mice^14^. CCL11 promotes cellular senescence by triggering pro-oxidant and pro-inflammatory pathways through SASP secretion^45^. When its levels decrease in old mice due to parabiosis with young counterparts, beneficial effects on long-term memory retention, spatial memory, hippocampal neurogenesis and local microglia activation occur^46^.

In a subsequent study by the same research group, β2M, a component of the major histocompatibility complex class I (MHC-I), was identified as an additional circulating factor that negatively regulates cognitive and regenerative functions in the adult hippocampus. Increased levels of β2M inhibit proliferation and differentiation of hippocampal neural progenitor cells, which affects cognition in mice. Notably, this adverse impact could be mitigated by reducing the expression of hippocampal transporter associated with antigen processing 1 (TAP1), which led to a decrease in cell MHC-I expression. This implies that the manifestation of β2M-induced deficits necessitates the presence of MHC-I on the cell surface^15^. Mechanistically, elevated levels of β2M may contribute to stabilizing interactions between MHC-I and paired immunoglobulin B-like receptor (PIRB) in mature hippocampal neurons. This interaction could potentially interfere with proper synaptic refinement, crucial for memory formation in adulthood^47^. Furthermore, β2M disrupts N-methyl-D-aspartate (NMDA) signaling by interacting with the S2 loop of GluN1^48^. NMDA signaling is essential for neuronal plasticity, as well as for learning and memory processes^49^.

Exposure of aged mice to a platelet-enriched plasma fraction from young mice produced a marked reduction in neuroinflammation in the hippocampus, both at the molecular and cellular levels. This study reported that exposure improved cognitive impairments associated with hippocampal function, as well as implicated platelet factor 4 (PF4) in the observed benefits. Systemic administration of PF4 to aged mice further demonstrated its potential to alleviate age-related neuroinflammation in the hippocampus, trigger molecular changes related to synaptic plasticity, and improve cognitive function. These favorable results of systemic PF4 in the aging brain are thought to be due to the reduction of circulating levels of pro-aging immune factors in the blood and restoration of the aging peripheral immune system. In addition, mechanistic insights revealed that the C-X-C chemokine receptor type 3 (CXCR3) plays a key role in mediating the cellular, molecular, and cognitive benefits associated with systemic PF4 administration in the aged brain^50^.

In contrast, exposing an aged animal to young blood can mitigate and even reverse certain effects of brain aging. For example, it increased dendritic spine density in mature neurons and enhanced synaptic plasticity in the hippocampus of aged heterochronic parabionts. At the cognitive level, age-related cognitive impairments showed improvements in both contextual fear conditioning and spatial learning and memory tasks. Structural and cognitive improvements observed in aged mice exposed to young blood are attributed, in part, to activation of cyclic AMP response element binding protein 1 (CREB1) in the aged hippocampus^51^. CREB1 is a key transcription factor that plays a crucial role in the regulation of genes associated with aging. One of the genes regulated by CREB1 is Ataxia Telangiectasia mutated (ATM), which is involved in several aspects of aging and DNA damage response^52^. A murine model of Huntington’s disease demonstrated that heterochronic parabiosis produces improvements associated with factors found in the serum exosome, although the specific components remain unidentified. These factors facilitate the activation of CREB1 and PGC1α, thereby inhibiting apoptosis and improving mitochondrial function^53^.

### Bone

Aging is a widely recognized risk factor associated with reduced bone density and deterioration of bone quality, resulting in increased susceptibility to fractures. Bones, as dynamic living tissues, undergo a perpetual cycle of formation and decomposition. This dynamic process is orchestrated by two types of cells, osteoblasts, which promote bone formation, and osteoclasts, which facilitate bone resorption. Maintaining a delicate balance between these activities is crucial to preserving optimal bone health^54^.

**Fig. 2:**
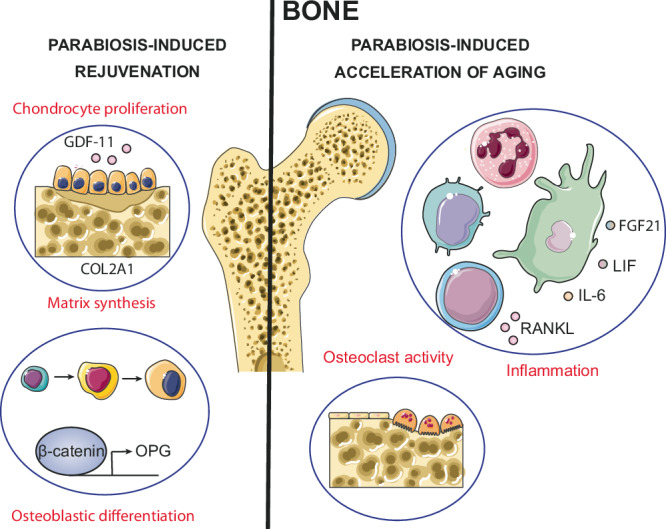
Effects observed in bone during heterochronic parabiosis. In the rejuvenation setting, there is an increase in chondrocyte proliferation, osteoblastic differentiation and subsequent matrix synthesis. Conversely, in accelerated aging scenarios, there is an increase in inflammation and osteoclast activity.

Heterochronic parabiosis exerts various effects on bone, affecting factors such as bone density, microarchitecture and remodeling processes (Fig. 2). One study reported that exposure to blood from aged mice markedly promoted osteoclastic activity, reduced bone mineral density and skeletal stem cell abundance in young heterochronic mice^27^. In contrast, increased chondrocyte proliferation and matrix synthesis were observed in cartilage from old mice exposed to a systemic milieu of a young mouse. This phenomenon was related to the action of growth differentiation factor 11 (GDF11), which attenuates age-related knee degeneration and promotes chondrocyte proliferation in the cartilage of old mice. GDF11 achieves these effects by up-regulating the SMAD2/3 pathway and promoting type II collagen (COL2A1) secretion^55^.

Heterochronic parabiosis has been shown to reverse the impaired fracture repair phenotype observed in aged animals, effectively restoring their diminished capacity for osteoblastic differentiation. This rejuvenating effect is facilitated by hematopoietic cells, which modulate the β-catenin signaling pathway during the initial stages of the repair process^56^. β-catenin triggers osteoprotegerin (OPG) transcription in osteoblasts. OPG is a cytokine receptor within the tumor necrosis factor (TNF) receptor superfamily. Its main function is to safeguard bone by binding to receptor activator of NF ligand (RANKL), thus preventing excessive bone resorption. This mechanism promotes osteoblast differentiation and helps maintain bone density^57,58^. However, with aging, β-catenin levels are elevated in the early phase of repair, and its reduction improves the quality of fracture healing^56^. In particular, young macrophage cells secrete lipoprotein receptor-related protein 1 (LRP1), a crucial molecule for reducing β-catenin signaling, thereby enhancing fracture repair in old bone marrow stromal cells^59^.

Another study revealed that when aged mice were exposed to the juvenile circulation by heterochronic parabiosis, there was a marked increase in the neutrophil population, especially CD11b + Ly6C + Ly6G++ neutrophils. This increase was accompanied by a corresponding increase in the levels of plasma extracellular vesicles associated with these neutrophils. It is believed that binding of the lymphocyte antigen 6 complex of the G6D locus (Ly6G+) to its ligand on neutrophils may exert an inhibitory effect on local immune responses. Consequently, these changes have been found to be related to the enhanced fracture healing observed in aged mice participating in heterochronic parabiosis couples^60^.

In a murine model of Duchenne muscular dystrophy (DMD) subjected to heterochronic parabiosis, remarkable effects were observed. Individuals with DMD often experience reduced bone density and weakened bones. In particular, older heterochronic mice exposed to blood from young mice experienced a significant reduction in trabecular bone loss and improved healing of bone lesions in the proximal tibia. However, these positive results were accompanied by detrimental changes in bone health observed in young mice exposed to blood from old mice, although to a lesser extent. The benefits observed in the older mice were potentially associated with an increase in the number of bone marrow hematopoietic stem cells and a reduction in cytokines such as fibroblast growth factor 21 (FGF21), leukemia inhibitory factor (LIF), IL-6, myostatin and RANKL^30^. All of these cytokines promote bone resorption through osteoclast activity and contribute to the creation of an inflammatory environment through various mechanisms^61–65^.

### Muscle

**Fig. 3:**
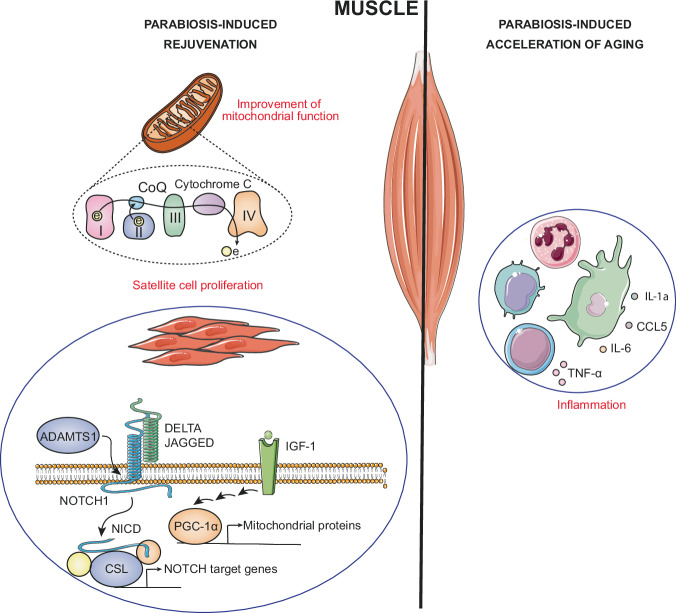
Effects observed in muscles during heterochronic parabiosis differ based on whether rejuvenation or accelerated aging is induced. In rejuvenation scenarios, there are enhancements in mitochondrial activity and stimulation of satellite cell proliferation for repair. Conversely, in accelerated aging contexts, there’s an increase in inflammation.

Heterochronic parabiosis exerts various effects on muscle cells (Fig. 3). A study found that numerous factors present in the juvenile circulation contribute to improved health of aging tissues. Specifically, some of these factors are thought to enhance myogenic proliferation by activating insulin-like growth factor 1 (IGF-1) and NOTCH signaling pathways, such as LIF. Others, such as cerberus (CER1), CRIPTO and dickkopf-related protein 1 (DKK1), counteract the TGF-β or WNT pathways, which usually impede the repair of aged muscle. Another subset of young proteins identified improving old muscle comprises tissue remodeling factors such as metalloproteinases and their inhibitors. Notable examples are tissue inhibitor of metalloproteinases 2 (TIMP2), cadherin-5 (CDH5) and vascular cell adhesion protein 1 (VCAM1), which facilitate cell-cell interactions. In addition, growth/differentiation factor 5 (GDF5) plays a role in regulating reinnervation, while factors involved in blood coagulation and vascular remodeling, such as serum amyloid protein A-1 (SAA1), fractalkine (CX3CL1), collagen α-1(XVIII) chain (COL18A1) and thromboplastin, are also found in this subset. Finally, leukocyte-specific proteins such as interleukin-27 (IL27), interleukin-10 (IL10), interleukin-22 (IL22), interleukin-22 receptor subunit α2 (IL22RA2) and lymphotoxin α (LTA) contribute to adult myogenesis^66^.

Satellite cells isolated from old mice subjected to heterochronic parabiosis with young mice showed enhanced colony-forming myogenic capacity and restored genomic integrity. GDF11 was also associated with these benefits. In addition, administration of this protein by injections to aged mice produced several positive results: restoration of myofiber size in regenerated muscle, increased mean size of regenerated myofibers, improved mitochondrial morphology, elevated levels of PGC1α, a key regulator of mitochondrial biogenesis, promotion of autophagy and mitophagy, improved regenerative potential of satellite cells, and increased neuromuscular junction size^67^. Although these findings are noteworthy, it is important to approach them with caution, as there are studies suggesting contrary effects of GDF11 on muscle. Some research indicates that GDF11 may hinder, rather than enhance, muscle regeneration by activating SMAD2/3 signaling and promoting inhibition of the differentiation process^68^.

In another study, it was observed that exposure of satellite cells from old mice to serum from young mice increased the activity of the NOTCH pathway and thus enhanced their proliferation^69^. Many components of the canonical NOTCH signaling pathway are known to play important roles in skeletal muscle development or function. Although the exact mechanisms by which this signaling pathway regulates muscle stem cells (MuSCs) are not fully understood, a crucial component appears to be ADAM metallopeptidase with thrombospondin type 1 motif 1 (ADAMTS1). This protein, secreted by macrophages, targets NOTCH1, thereby stimulating MuSC activation and promoting muscle regeneration^70^. High testosterone levels in the young mouse have also been identified as a stimulator of this signaling pathway in the old mouse after heterochronic parabiosis, playing a crucial role in the improvement of muscle mass and ultrastructure^71^.

In contrast to previous findings, exposure of a young mouse to blood from an older mouse leads to a decrease in muscle functional performance, indicating detrimental effects of circulating factors^72^. Mitochondria play a fundamental role in the physiology of skeletal muscle, as they are responsible for regulating the metabolic state of the tissue and providing the energy necessary for its correct functioning^73^. In this sense, muscle samples obtained from young heterochronic mice showed significant reductions in both total mitochondrial area and mean mitochondrial size. In addition, a trend toward decreased expression of mitochondrial complex IV was observed. Furthermore, there was a marked decrease in oxygen consumption and activity of mitochondrial complexes I, II and coenzyme Q10 (CoQ10), as well as the entire electron transport system (ETS) in general^72^.

The systemic milieu of an aged mouse triggers an increase in SASP factors in a young mouse, including IL-1a, IL-6, CCL5 and TNF-α. In this manner, the inflammatory process induced by these circulating factors can trigger tissue dysfunction. These factors primarily affect skeletal muscle, but also other organs such as the kidneys and liver. These circulating factors cause a decrease in muscle strength, physical endurance and lipid accumulation in muscle tissue. They also induce an increase in senescent satellite cells and senescent muscle interstitial cells in young mice. However, these effects can be mitigated by pre-treating the older mouse with senolytic drugs such as dasatinib plus quercetin or navitoclax before subjecting it to heterochronic parabiosis^26^.

miR-199-3p is significantly decreased in the blood of aged mice compared to young mice. Restoration of its plasma levels by heterochronic parabiosis enhances myogenic differentiation and muscle regeneration by targeting LIN28 homolog B (LIN28B) and suppressor of zeste 12 protein homolog (SUZ12). This, in turn, enhances the expression of myogenic differentiation 1 (MYOD1) and myogenic factor 5 (MYF5)^74^.

### Hematopoietic and immune cells

**Fig. 4:**
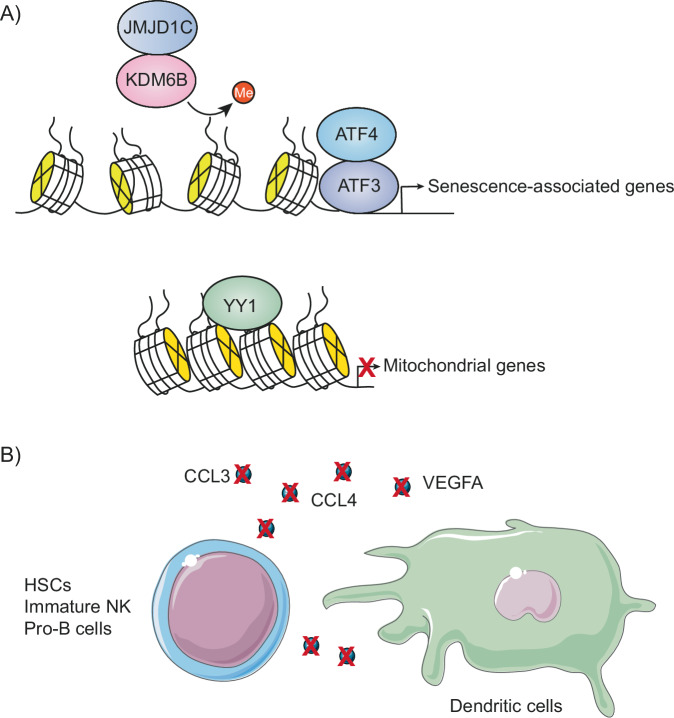
Detrimental effects of parabiosis-induced accelerated aging on hematopoietic and immune cells. **A** Increased expression of senescence-associated genes in blood cells, along with reduced expression of mitochondrial genes. **B** Disruption of intercellular communication between immune cells.

A study focused on hematopoietic and immune cells found that they exhibit considerable transcriptional plasticity in the context of heterochronic parabiosis (Fig. 4). This flexibility is particularly evident in hematopoietic stem and progenitor cells, which encompass both short- and long-term self-renewing hematopoietic stem cells and multipotent progenitor cells. Up-regulated genes associated with parabiosis-accelerated aging were consistently associated with neutrophil activation and antimicrobial response, whereas down-regulated genes were associated with reduced cytokine production, hematopoiesis, chromatin organization, circadian rhythm regulation, and cell cycle regulation. In contrast, parabiosis-induced rejuvenation produced opposite effects on these gene expressions^75^.

The observed changes were attributed to several factors. These included transcription factors such as activating transcription factor 3 (ATF3) and activating transcription factor 4 (ATF4), as well as chromatin organization and remodeling factors such as lysine-specific demethylase 6B (KDM6B), Jumonji domain-containing protein 1C (JMJD1C) and yin and yang 1 (YY1)^75^. ATF3 and ATF4 play a vital role in inducing specific senescence-associated mRNAs, either by directly acting on their promoters or by modulating chromatin accessibility^76,77^. Meanwhile, KDM6B plays a key role in the aging process by modulating several signaling pathways. It regulates the expression of factors involved in key cellular processes such as CDKN2A, TGF-β/SMADs and SASP factors. These factors act as transcriptional regulators, inhibiting or activating gene expression to promote cell cycle arrest and other aging-related changes^78,79^. YY1 recruits sirtuin 6 (SIRT6) to the transcription factor A, mitochondrial (TFAM) gene via YY1-associated factor 2 (YAF2), which ultimately contributes to the decline in mitochondrial function associated with aging^1,80^.

In addition, parabiosis-accelerated aging was observed to affect cell-cell communication pathways involving cytokine-cytokine receptor interactions, in particular CCL3, CCL4 and VEGFA. However, exposure to young blood restored these pathways. Especially striking was the rejuvenation-induced reestablishment of communication between hematopoietic stem cells, dendritic cells, immature NK cells and pro-B cells, which had been disrupted during aging^81^. These dysfunctions in intercellular communication play an important role in the aging process. They contribute to the chronic inflammation, as well as to deficiencies in the immune system’s ability to detect and eliminate pathogens and precancerous cells^4^.

Heterochronic parabiosis did not enhance the cellularity of peripheral lymph nodes of old mice exposed to the systemic milieu of young mice. This suggests that the environment itself limits cellularity in these organs. Interestingly, the T cells in the peripheral lymph nodes of these old animals showed a higher proportion of self T cells with a peripheral maintenance advantage than T cells of the young mouse^82^.

Heterochronic parabiosis experiments demonstrated that the young systemic milieu only partially corrected the abnormalities of erythrocyte metabolism in old mice. Specifically, it normalized methionine levels and elevated erythrocyte choline levels^83^. Methionine enhances antioxidant capacity in erythrocytes, while choline enhances erythrocyte deformability, reduces erythrocyte aggregation, and increases membrane lipid fluidity^84,85^. In addition, parabiosis reduced levels of early glycolytic metabolites in erythrocytes and partially restored levels of late glycolytic products (pyruvate and lactate) to those observed in young mice. However, these interventions showed minimal or no effect on erythrocyte glutathione homeostasis, the pentose phosphate pathway, and the oxidation of purines and tryptophan, which are all associated with maintaining redox balance^83^.

### Liver

**Fig. 5:**
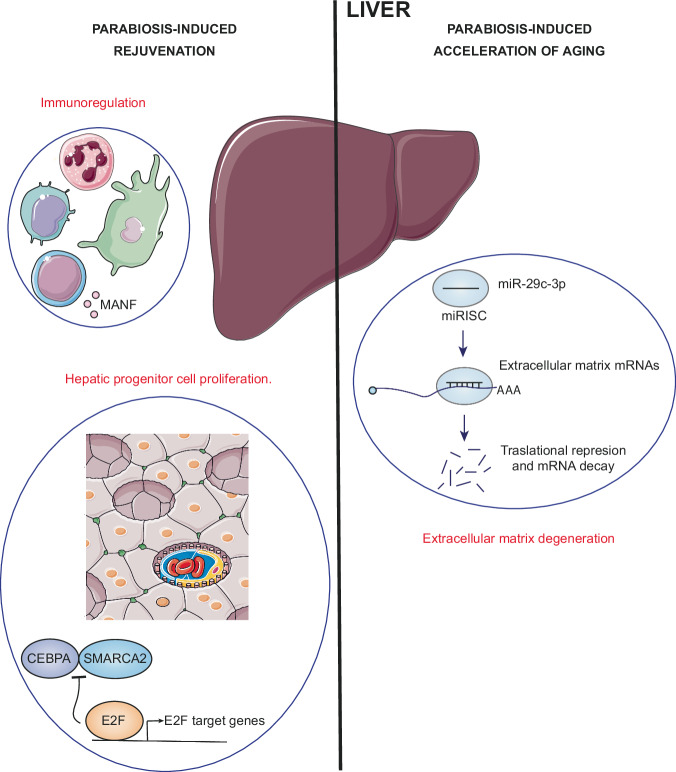
Effects observed in the liver during heterochronic parabiosis. From the perspective of parabiosis-induced rejuvenation, improvements in immunoregulation are observed and proliferation of hepatic progenitor cells is promoted. In contrast, in the context of parabiosis-induced accelerated aging, degradation of extracellular matrix-associated transcripts occurs.

Heterochronic parabiosis exerts various effects on hepatocytes (Fig. 5). Heterochronic parabiosis elevated the proliferation of aged hepatic progenitor cells by reducing the levels of CCAAT/enhancer-binding protein α (CEBPA)-SWI/SNF-related matrix-associated actin-dependent regulator of chromatin subfamily A member 2 (SMARCA2) complex to levels comparable to those seen in young animals, thereby facilitating E2F-driven gene expression^69^. Aging leads to elevated levels of SMARCA2, which interacts with CEBPA, initiating the formation of the CEBPA- retinoblastoma-associated protein (RB1)-E2F transcription factor 4 (E2F4) complex. This complex binds to E2F-regulated promoters, suppressing the expression of their target genes. Consequently, it inhibits MYC promoter activation, causing a decrease in the proliferative response^86^.

Notably, mesencephalic astrocyte-derived neurotrophic factor (MANF), a stress response protein secreted by cells with immunomodulatory properties, is also important for rejuvenating the liver by heterochronic parabiosis. After parabiosis-induced rejuvenation, MANF is crucial for decreasing markers of inflammation, reducing the accumulation of fibrotic areas and preventing hepatocyte apoptosis in the liver. However, it does not have the ability to reverse hepatosteatosis. These effects are closely linked to an enrichment of circadian rhythm and metabolic pathways^87^.

In mice undergoing heterochronic parabiosis-induced rejuvenation, levels of miR-29c-3p, a non-coding RNA (ncRNA) strongly associated with aging in various organs, plasma, and extracellular vesicles (EVs), were significantly decreased. This reduction brought its levels close to those observed in the liver of young mice. Specifically, miR-29c-3p targets genes related to the extracellular matrix and secretory pathways, such as elastin (ELN), collagen α1(I) chain (COL1A1), collagen α2(I) chain (COL1A2), collagen α1(III) chain, disintegrin and metalloproteinase domain-containing protein 12 (ADAM12) and ADAM metalloproteinase with thrombospondin motifs 17 (ADAMTS17), all of which play roles in the aging process^8^.

Exposure of young animals to the systemic environment of old mice resulted in a rapid decline in hepatic functional performance. Moreover, when muscle injury was introduced into these animals, these detrimental effects were further exacerbated^28^. In addition, these mice also exhibit hepatic fibrosis, highlighting the impact on several systems^26^.

### Heart

After 4 weeks of exposure to the circulation of young mice, a notable regression of cardiac hypertrophy was observed in old mice. This regression was characterized by a reduction in the size of cardiomyocytes and molecular remodeling. Specifically, there was a decrease in the expression of atrial natriuretic peptide (ANP) and brain natriuretic peptide (BNP), along with an increase in levels of sarcoplasmic/endoplasmic reticulum calcium ATPase 2 (SERCA2)^88^. Cardiac natriuretic peptides, such as ANP and BNP, are vital for maintaining cardiovascular balance and heart health. They not only influence blood pressure regulation, but also the management of glucose and lipid metabolism^89^. Meanwhile, SERCA2a facilitates the movement of calcium ions (Ca^+2^) from the cytoplasm to the sarcoplasmic reticulum, a process crucial for myocardial relaxation. This mechanism directly influences the heart’s ability to contract efficiently and maintain a regular rhythm, essential for overall cardiac function. With aging, the regulation of SERCA2a activity can become dysregulated, potentially contributing to age-related changes in cardiac performance and the development of cardiovascular conditions^90^.

Furthermore, young cardiosphere-derived extracellular vesicles induce structural and functional improvements in the hearts, lungs, skeletal muscles, and kidneys of old rats. Indeed, these vesicles positively modulate glucose metabolism and activate anti-senescence pathways, contributing to overall rejuvenation^91^.

One study reported that GDF11, as well as its effects on other tissues, plays a role in mediating the positive changes elicited by heterochronic parabiosis. This protein is abundant in the blood of young mice and decrease with age. Research showed that when its levels are increased in old mice subjected to heterochronic parabiosis, it contributes to reversing cardiac hypertrophy^88^. However, a subsequent study failed to replicate the impact on cardiac hypertrophy observed previously and found minimal effects of GDF11 injections^92^. Significantly, there were notable discrepancies between the two studies, including variations in the origin and sex of the mice used, as well as differences in the source of the recombinant active domain of GDF11 administered in the injections. A third study shed new light on the matter, revealing that overexpression of GDF11 led to a reduction of senescent cells in the hearts of aged mice. Moreover, it stimulated cardiac stem cell proliferation and facilitated the recruitment of endothelial progenitor cells, ultimately resulting in increased angiogenesis in aged ischemic hearts^93^. Benefits were noted in mitigating age-related cardiac fibrosis and enhancing cardiac pump function under pressure overload conditions. Nevertheless, it was also noted that administering high doses of GDF11 could lead to severe cachexia and mortality^94^.

### Vascular endothelium

Parabiosis has been shown to improve vascular endothelial dysfunction in aged female mice. In the group of aged mice subjected to heterochronic parabiosis, there was a marked decrease in the maximal relaxation response to acetylcholine during the third week. However, this effect was no longer evident by the ninth week after the parabiosis protocol^95^. This observation suggests that the improvement in vascular function may not be sustained over time, possibly indicating that the circulating factors responsible for the improvement lose their efficacy over time.

Blood from young mice markedly enhanced the ability of blood vessels from old mice subjected to heterochronic parabiosis to relax in response to endothelial signals, and also reduced the production of ROS. These effects appeared to be mediated by a number of protective effects on mitochondria. These effects included improvements in oxidative phosphorylation and the tricarboxylic acid cycle, as well as anti-inflammatory and antioxidant effects^96^.

Contrary to earlier findings, genes affected by old mouse blood in young parabiont mice could potentially play a role in inducing pathological changes in the vascular structure of the aortic arch. This could occur through the suppression of pathways controlled by serum response factor (SRF), insulin-like growth factor 1 (IGF-1), and vascular endothelial growth factor A (VEGF-A)^97^. SRF-associated genes encode proteins essential for processes such as proliferation, repair and regeneration in vascular endothelial cells, which are critical for maintaining the health and function of blood vessels^98^. Meanwhile, IGF-1 plays a protective role against various cardiovascular conditions, including endothelial dysfunction and atherosclerotic plaque formation^99^. Lastly, VEGF is an important growth factor known for its crucial proangiogenic properties. It exerts mitogenic and antiapoptotic effects on endothelial cells, stimulating cell proliferation and preventing cell death. In addition, VEGF increases vascular permeability and facilitates cell migration, processes essential for angiogenesis and tissue repair^100^.

### Kidney

Parabiosis-induced acceleration of aging triggers various adverse effects in the kidneys, including necrosis of tubular epithelial cells, interstitial inflammation, and abnormalities in the glomeruli^26^. In another study, researchers observed a decrease in renal tubular degeneration and the number of tubular cell detachments, along with reductions in markers of senescence and inflammation in aged mice. Furthermore, there was an increase in autophagy factors and antioxidant markers in the kidneys. These findings underscore the significant mitigation of renal aging achieved by exposure to a young blood environment^101^.

### Eyes

A study on lacrimal glands revealed that young mice exposed to the systemic milieu of older mice exhibited increased lymphocytic infiltration, especially of marginal zone B cells and plasma cells. In addition, there was an increase in inflammatory cytokines such as interferon-γ (IFNγ), IL1b and C-X-C motif chemokine 9 (CXCL9), along with elevated focal scores indicating the severity of inflammation. Interestingly, male mice showed more pronounced effects than females during heterochronic parabiosis. In contrast, aged mice exposed to the systemic milieu of their young counterparts showed minimal improvements in this tissue^25^.

Studies of accelerated aging by parabiosis have shown the role of tropomyosin α1-chain protein (TPM1) in the regulation of age-related inflammatory responses, glial cell activation, and abnormal dendrite sprouting in the aged retina. This regulation involves activation of protein kinase A (PKA) signaling and subsequent modulation of the fatty acid-binding protein 5 (FABP5)/NF-κB signaling pathway. Further heterochronic parabiosis experiments with old mice lacking TPM1 protein, which did not induce similar effects in young mice, supported this role^102^.

### Visceral adipose tissue (VAT)

In a heterochronic parabiosis model of induced rejuvenation, a significant reduction of proinflammatory cytokine levels and an alteration of the adipokine profile was observed in the VAT of old mice exposed to blood from young mice. This alteration of the adipose tissue microenvironment was found to be protective against cellular senescence. Cells of the stromovascular fraction derived from aged adipose tissue showed reduced production of proinflammatory cytokines such as CCL2 and IL-6. In addition, reduced expression of senescence-related markers such as CDKN2A and CDKN1A was observed. Furthermore, levels of senescence-related adipokines, such as C-reactive protein (CRP), endothelial cell-specific molecule 1 (ESM1), intercellular adhesion molecule 1 (ICAM1), IL-11, leptin, pentraxin 2, and serpin E1/plasminogen activator inhibitor 1 (PAI-1), were reduced in old mice exposed to blood from young mice. These results demonstrate that the young milieu has the ability to protect aged adipose tissue from senescence and inflammation^103^.

One study identified 233 uniquely deregulated miRNAs in different organs associated with induced rejuvenation and 43 with accelerated aging, mainly in gonadal and mesenteric adipose tissues^8^. Another study found that upregulation of miR-25-93-106b in old mice due to parabiosis resulted in decreased fat mass by reducing SIRT7 expression^104^. SIRT7 is a key regulator of adipogenesis, promoting differentiation and maturation of early adipocyte precursors^3^.

### Other tissues

The thymus serves as a primary lymphoid organ crucial for the maturation of T lymphocytes, a key component of the immune system. However, as individuals age, the thymus undergoes a process known as thymic involution, which is characterized by a decline in its functionality and size. This age-related decline in thymic function is a significant aspect of immune senescence, contributing to a decline in immune responsiveness with age^105^. Despite attempts with heterochronic parabiosis, thymic involution proved irreversible^82,106^.

When young mice were paired with old mice, significant elevations were observed in the levels of the intervertebral disc markers matrix metalloproteinase-13 (MMP13) and ADAM metalloproteinase with thrombospondin motifs 4 (ADAMTS4), as well as in aggrecan core protein (ACAN) fragmentation and histological tissue degeneration. However, there were negligible changes in cellular senescence markers such as CDKN2A and CDKN1A. The result was accelerated disc matrix imbalance and tissue degeneration, with minimal impact on disc cellular senescence. In contrast, when old mice were paired with young mice, the effects were opposite. This pairing significantly suppressed disc cellular senescence but produced only a slight decrease in disc matrix imbalance and degeneration^107^.

As people age, the accumulation of senescent cells increases, contributing to pancreatic β-cell dysfunction and insulin resistance in peripheral tissues. These effects play an important role in the development of type II diabetes mellitus^108^. Old mice parabiosed to young mice show increased pancreatic β-cell replication. In contrast, young β cells transplanted into old mice decrease their replication rate^109^.

## Conclusions and future perspectives

Heterochronic parabiosis has proven to be a valuable tool to decipher some key circulating molecules involved in the aging process, both promoting and delaying it. In general, circulating factors exchanged during parabiosis may promote or delay cellular senescence and help eliminate senescent cells. Parabiosis also appears to rejuvenate mitochondrial function in several contexts. Parabiosis has also been shown to regulate inflammatory processes, either by promoting them during accelerated aging or by preventing them during induced rejuvenation. Specifically, in the brain, accelerated aging leads to altered intercellular communication and increased DNA damage culminating in genetic instability. In contrast, induced rejuvenation enhances proteostasis and epigenetic modifications. In bone marrow, muscle and liver, stem cell depletion is mitigated during induced rejuvenation. Mitochondrial function is improved in brain, hematopoietic and immune cells, vascular endothelium and muscle. In addition, macroautophagy plays a crucial role in muscle and kidney rejuvenation. Accumulation of senescent cells in the brain, pancreas, hematopoietic and immune cells, muscle and VAT is prevented during induced rejuvenation. Chronic inflammation is favored during accelerated aging by parabiosis in the brain, bones, muscles, liver, vascular endothelium, kidneys, eyes and VAT. In contrast, induced rejuvenation reduces inflammation in these tissues and organs.

Proteins identified as relevant in the aging process through this strategy are poised to be prominent targets, first to better understand this intricate process and then to elucidate strategies to delay the harmful effects of aging, such as various age-related diseases, thus improving our quality of life. Researchers around the world can search for inhibitors for targets that promote aging and activators for those that delay it. This is of great relevance given that currently 8.5% of people worldwide (617 million) are 65 years of age or older, and it is estimated that by 2050 this figure will triple, representing almost 17% of the world’s population (1.6 billion)^110^.

It is important to note that one of the main challenges faced by studies on heterochronic parabiosis is that, in most cases, the conditions used vary significantly (Table 1). This includes variations in factors such as the sex and age of the animals, their location and cross-linked blood vessels, the length of the cross-linking period, surgical procedures, diet and exercise capacity, among other variables. Therefore, it would be appropriate to establish a convention where the conditions are the same or as similar as possible in order to enhance reproducibility.

Comparing the benefits of induced rejuvenation with the deleterious effects of accelerated aging is complex, as the changes do not usually occur in opposite directions within the same processes. On the contrary, they often involve changes in the same direction, possibly indicating repair, compensatory mechanisms, or alterations in entirely different processes. Despite this complexity, it would be valuable to find a method to evaluate these effects, possibly using statistical and computational models. Additionally, studies aimed at determining whether the age difference between animals and the duration of their cross-linking lead to significant variations in heterochronic parabiosis outcomes would be particularly insightful.

Also, it should also be noted that most studies focus mainly on circulating proteins, while information on other circulating biomolecules such as DNA, non-coding RNAs, extracellular vesicles, lipids, carbohydrates and their metabolites is rather limited. Similarly, most research only considers blood cells, despite the fact that other cell types can be transferred. This disparity underscores the need for further research to better understand the roles and mechanisms of these less studied biomolecules and cells in various biological processes.

Exploring the combined dataset of transcriptomic, epigenomic, proteomic and metabolomic information derived from various tissues and cells in parabiosis experiments has the potential to provide a holistic understanding of the aging process^111^. This integrated approach could unveil intricate molecular mechanisms underlying aging-related changes, providing a comprehensive and structured view of how different biological pathways interact and contribute to aging. However, the analysis of these multidimensional data sets presents significant challenges due to their complexity and the large amount of information they encompass. This complexity is due to the interaction of various molecular processes and the need to integrate data from different omics levels. To meet this challenge, new computational methods and artificial intelligence techniques adapted to big data analysis and pattern recognition can be of great value^112^. These advanced tools can help identify key molecular signatures, extract meaningful insights and uncover hidden associations in the data, thus facilitating a deeper understanding of the biology of aging and the development of potential interventions to promote healthy aging.

There are still many challenges and opportunities to be explored with heterochronic parabiosis. Among them, standardizing protocols to obtain as much information as possible and ensure reproducibility, identifying more specific factors with pharmaceutical potential, defining how transferable the findings are to humans, among many other things. It would be interesting if similar experiments could be carried out in long-lived rodents, such as naked mole rats^113,114^ or blind mole rats^115^, as well as in other mammalian models of aging, such as bats^116^, or in animals that experience a rapid decline in health leading to death, such as boreal quolls during the breeding season^117^. These studies could provide valuable information, especially when compared to findings in mice.

Considering that a natural linkage similar to heterochronic parabiosis occurs during pregnancy^118,119^, it raises intriguing questions about its effects. Specifically, it raises the analysis of whether the mother benefits from circulating factors shared with the baby, while the baby might experience adverse effects from factors present in the mother’s blood. In a recent article, it was pointed out that pregnancy accelerates the mother’s aging process at the biological level. However, the act of giving birth seems to have a counteracting effect, effectively slowing down this aging process^120^. Understanding the regulation of this phenomenon, even if no such effects occur, is of great interest.

The use of senolytics to target and eliminate senescent cells is currently being thoroughly investigated. Drugs such as dasatinib - approved to treat certain types of cancer - and quercetin - marketed as an antioxidant dietary supplement - are known to reduce age-related acceleration of senescence. Numerous clinical trials are testing their effects in a variety of age-related diseases. Investigation of these treatments in the context of heterochronic parabiosis could provide valuable data. For example, examining whether the beneficial effects of young blood on aging tissues are enhanced by senolytics, or whether the detrimental effects of old blood on young tissues are mitigated. Technologies such as single-cell RNA sequencing could allow us to analyze in detail the effects on different cell types, providing a deeper understanding of the mechanisms at play.
